# Recent advances in the role of selenium and nanoselenium in modulating plant defense under biotic and abiotic stresses

**DOI:** 10.3389/fpls.2026.1815681

**Published:** 2026-05-11

**Authors:** Fareeda Akhter, Shaista Rashid, Qamer Ridwan, Nahila Anjum, Durukhshan Zehra, Mohd Asgher, Manzoor R. Khan, Latif Ahmad Peer, Bilal Ahmad Mir, Mohd Hanief, Tanveer Alam Khan

**Affiliations:** 1 Department of Botany, Baba Ghulam Shah Badshah University, Rajouri, India; 2 Central University of Jammu, Jammu, India; 3 Department of Botany, North Campus, University of Kashmir, Baramulla, India; 4 Department of Botany, University of Kashmir, Srinagar, India; 5 Department of Biology, College of Science, United Arab Emirates University, Al Ain, United Arab Emirates

**Keywords:** abiotic stress, biotic stress, nanoselenium, selenium, synthesis

## Abstract

Climate change-driven abiotic and biotic stresses are emerging as a major threat to crop productivity and global food security. Consequently, there is a great need to develop effective and ecologically sustainable strategies to augment the growth, development, and yield of crop plants, especially under adverse environmental conditions. Nanoparticle-mediated precise modulation of soil-plant interactions has emerged as an eco-friendly, biocompatible, and stimulus-responsive strategy to increase crop production by ameliorating abiotic and biotic stresses. Selenium (Se), a trace element, has emerged as a promising mitigator of diverse stresses, including heavy metal toxicity, salinity, drought, pathogens, and pests. The use of selenium nanoparticles (SeNPs) has emerged as a potential strategy to enhance plant stress resilience, due to their increased biocompatibility, reduced toxicity, and greater stability, which provide advantages over inorganic forms of Se. SeNPs are readily absorbed by plants through root hairs and thereby improve plant growth, regulate physiological processes, stimulate antioxidants and redox balance, upregulate stress-responsive genes, and fortify stress tolerance mechanisms in plants. This review presents a comprehensive analysis of Se uptake pathways, its speciation, and incorporation into plant metabolic systems, as well as the diverse physiological and biochemical roles of Se and/or SeNPs in regulating plant defense mechanisms. A key focus is placed on SeNPs as a powerful tool for nano-enabled stress alleviation and biofortification in crop plants in modern agriculture. We further highlight how integrated multi-omics approaches are decoding the complex molecular networks underlying Se-mediated tolerance. However, the narrow optimal concentration window between benefit and phytotoxicity demands precise application. By bridging fundamental mechanisms with emerging nano-biotechnological applications, this review establishes the importance of Se and SeNPs as promising and sustainable, eco-friendly agents for developing climate-resilient crops, directly contributing to future food security.

## 1 Introduction

Fluctuating environmental conditions impose significant physiological stress on plants, disrupting growth and metabolic processes and ultimately reducing agricultural productivity (Hasanuzzaman et al., 2020a; Ahmad et al., 2021; Lanza and Reis, 2021; Moulick et al., 2024). Climate change, driven by increasing greenhouse gas concentrations, is further intensifying these stresses, contributing to documented declines in crop yield and quality (Muthamilarasan et al., 2019; Moulick et al., 2024; Wahab et al., 2024; Hultgren et al., 2025). Moreover, the rapid growth of the global population necessitates a sustained increase in agricultural production, placing additional pressure on the agricultural sector. Projections indicate that the global population may reach approximately 10 billion by 2050 and 11 billion by 2100, underscoring the urgent need to enhance agricultural land productivity (Sowmya et al., 2025). In addition, factors such as land degradation, shrinking arable land, and conventional abiotic stresses (e.g., drought, salinity, extreme temperatures, and heavy metals), along with the accumulation of emerging pollutants and biotic stresses, have led to a considerable reduction in crop yields worldwide (Benbrook et al., 2021; Sowmya et al., 2025), thereby necessitating innovative strategies to ensure food security. Additionally, the excessive and disproportionate application of chemical inputs, including fertilizers, pesticides, and insecticides, adversely impacts agricultural ecosystems by reducing soil fertility and biodiversity, and promoting pathogen resistance (Tsivileva, 2025). Addressing these critical challenges requires the development of innovative strategies that enhance crop resilience and minimize pressures on agricultural systems.

Among modern agro-technologies, the integration of nanotechnology into agriculture has emerged as a transformative approach, offering precisely engineered nanoparticles capable of re-orchestrating plant–environment interactions through stimuli-responsive behavior and size-dependent properties (Lowry et al., 2019; Qin et al., 2025). Nanomaterials are defined as engineered materials with at least one dimension less than 100 nm (Fatima et al., 2024). Commonly, these include nanocomposites, nanoparticles (NPs), nanofertilizers, nanorods, and nanodots (Paras et al., 2022). Owing to their nanoscale dimensions, nanomaterials exhibit unique physicochemical properties that facilitate intimate interactions within the soil–plant system. Particularly, nanomaterials in the size range of 5–20 nm can traverse plant cell walls and function as efficient carriers for nutrient uptake and delivery (Schwab et al., 2016; Arivalagan and Das, 2026). Furthermore, nanoparticles have been shown to enhance plant proliferation and propagation through improved callus development.

In this context, selenium (Se), a trace element, has gained prominence for its role in supporting plant growth and development and for its notable efficacy in mitigating a wide spectrum of biotic and abiotic stresses, including heavy metal toxicity, salinity, drought, extreme temperatures, and UV radiation (Feng et al., 2013; Hasanuzzaman et al., 2020a; Samynathan et al., 2023). The beneficial role of Se extends across various crop species, demonstrating its broad applicability (Hasanuzzaman et al., 2021). Although Se is not considered an essential element for most plants, its function as a potent plant protectant is well established (El-Ramady et al., 2016; Gupta and Gupta, 2016; Hasanuzzaman et al., 2020a). Se is widely recognized for enhancing plant growth, promoting photosynthesis, maintaining membrane integrity, boosting antioxidant capacity, and improving soil fertility, thereby strengthening plant responses to diverse environmental stresses (Lanza and Reis, 2021; Khan et al., 2023; Moulick et al., 2024).

Among promising nanoscale materials, selenium nanoparticles (SeNPs) have attracted considerable attention from plant scientists due to their multifunctional properties, such as nanoscale size, high compatibility with plant cells, strong antioxidant activity, enhanced bioavailability, low toxicity, eco-friendly nature, and cost-effectiveness, making them particularly attractive for agricultural applications compared with bulk selenium and other metallic nanoparticles (Ran et al., 2024a; Wahab et al., 2024; Sowmya et al., 2025). These characteristics enable the slow and controlled release of selenium while minimizing undesirable interactions with surrounding biomolecules (Skalickova et al., 2017). Furthermore, owing to these attributes, SeNPs are recognized as effective stress-mitigating agents and biostimulants that alleviate the adverse effects of abiotic stresses, such as heat, cold, heavy metals, salinity, and drought, as well as biotic stresses, thereby enhancing stress resilience in crop plants (Qin et al., 2025; Sowmya et al., 2025). However, excessive concentrations of SeNPs may induce phytotoxicity (Hussein et al., 2019). SeNPs modulate key physio-biochemical processes, including photosynthesis, nutrient acquisition, and phytohormone signaling, and enhance the scavenging of reactive oxygen species (ROS) through the upregulation of both enzymatic and non-enzymatic antioxidant defense systems. They also stimulate secondary metabolism, thereby reducing oxidative damage and ultimately improving plant growth and tolerance to multiple stress conditions (Cartes et al., 2010; Chauhan et al., 2019; Hussein et al., 2019; Garza-García et al., 2023).

The efficacy of Se in plants is intrinsically linked to its behavior within the soil–plant system. Its distribution varies across the biosphere, being present in the lithosphere, hydrosphere, pedosphere, and atmosphere (Floor and Román-Ross, 2012; White, 2018). Its bioavailability is primarily governed by chemical speciation and concentration, with soil pH playing a critical regulatory role (Zhang et al., 2014; Zhang and Chu, 2022). The uptake of Se occurs via sulfate transport pathways due to the chemical similarity of selenite (SeO_3_²^-^) and selenate (SeO_4_²^-^) to sulfate, with sulfate transporters primarily facilitating selenate uptake (Takahashi, 2019; Khan et al., 2022), followed by complex intracellular metabolism that determines its ultimate physiological effects (Freeman et al., 2010; White, 2018). SeNPs also play a significant role in enhancing plant tolerance to biotic stresses, such as pathogens and insect pests, by inducing structural and functional changes in soil microbial communities (Li et al., 2023). Se application enhances plant resistance to pathogen invasion and promotes soil microbial diversity, thereby improving photosynthesis, growth, and reducing oxidative stress (Hashem et al., 2022). The beneficial effects of Se and SeNPs under abiotic stress have been widely reported, including salinity stress in *Phaseolus vulgaris* (Admasie et al., 2023) and *Glycine max* (Wang et al., 2023a), drought stress in tomato (*Solanum lycopersicum*) (Neysanian et al., 2020) and *Triticum aestivum* (Hasanuzzaman et al., 2024), cadmium stress in *Solanum lycopersicum* (AlYemeni et al., 2018; Ahmad et al., 2024), and heat stress in *Cucumis sativus* (Balal et al., 2016), where they enhance stress tolerance. Additionally, soil-applied Se can inhibit growth and reduce the relative abundance of pathogenic fungal communities (Li et al., 2023). Moreover, SeNPs have been reported to be more effective than bulk Se in suppressing pathogen growth and abundance (Zohra et al., 2021; Li et al., 2023).

The synthesis of selenium nanoparticles (SeNPs) can be broadly categorized into top-down (physical) and bottom-up (chemical and biological/green) approaches (Sowmya et al., 2025). Physical methods include photothermal-assisted synthesis, microwave-mediated synthesis, electrodeposition, and pulsed laser ablation (Burmistrov et al., 2025). Chemical methods involve the reduction of inorganic Se precursors followed by surface modification using a diverse range of stabilizing and capping agents, such as folic acid, ascorbic acid, gallic acid, and benzoic acid, in aqueous solutions (Skalickova et al., 2017). Among these approaches, green synthesis using plant extracts has gained particular attention due to its eco-friendly nature, cost-effectiveness, and potential to enhance the bioactivity of SeNPs (Zohra et al., 2021). Se/SeNPs are readily taken up by plants through roots via sulfate and phosphate transporters and subsequently translocated to the shoots. Following uptake, SeNPs enter the vascular system and are transported to aerial tissues through transpiration-driven flow and nutrient transport pathways. Within plant tissues, Se and SeNPs undergo biotransformation and are assimilated via sulfur metabolic pathways into organic selenium compounds, such as selenomethionine (SeMet) and selenocysteine (SeCys), which are subsequently incorporated into proteins and other biomolecules (Zhou et al., 2021; Arivalagan and Das, 2026).

Se and SeNPs play a significant role in improving plant health and productivity through multiple physiological and molecular mechanisms. The beneficial effects of Se in plants can be broadly grouped into five major categories: (1) enhancement of growth, biomass, and yield; (2) maintenance of antioxidants and redox homeostasis; (3) improvement of stress tolerance; (4) regulation of physiological and biochemical processes; and (5) modulation of soil microbial diversity to support plant growth. At optimal concentrations, Se enhances photosynthetic efficiency, stabilizes chlorophyll, and improves nutrient uptake, thereby promoting plant growth, biomass accumulation, and crop yield. In addition, Se plays a crucial role in maintaining cellular redox homeostasis by regulating reactive oxygen species (ROS) signaling and activating antioxidant defense systems, including superoxide dismutase, catalase, thioredoxin reductase, and glutathione peroxidase, which collectively protect plants from oxidative damage (Wang et al., 2007; Mittler, 2017; Hasanuzzaman et al., 2020a; Alshaal et al., 2025). Selenium also contributes to physiological and biochemical regulation by supporting chloroplast functionality, modulating metabolic pathways, and preserving membrane integrity. Furthermore, Se application can reshape rhizosphere microbial communities, enhancing nutrient cycling and plant–microbe interactions that promote sustainable plant growth (Arivalagan and Das, 2026). Beyond these general roles, Se and SeNPs modulate specific defense mechanisms that strengthen plant tolerance to both biotic and abiotic stresses. For instance, SeNP supplementation has been shown to increase cell wall thickness by elevating pectin and hemicellulose content, thereby reinforcing structural barriers against environmental stress and pathogen invasion (Alshaal et al., 2025). Moreover, Se and SeNPs enhance plant defense responses by regulating phytohormone signaling, activating defense-related enzymes, and strengthening antioxidant systems, which collectively reduce oxidative damage and improve resilience to diverse stress conditions (Sowmya et al., 2025; Narang et al., 2025; Muthamilarasan et al., 2019; Yang et al., 2021; Arivalagan and Das, 2026). Furthermore, plant physio-biochemical processes are intricately controlled at the molecular level through gene expression and signaling networks. Stress conditions induce dynamic changes in the expression of genes associated with cellular metabolism, growth, and defense responses, thereby influencing overall plant performance. In this context, the integration of multi-omics approaches, including genomics, transcriptomics, proteomics, and metabolomics, has emerged as a powerful strategy to enhance plant stress tolerance. These approaches provide comprehensive insights into regulatory networks, genes, proteins, and metabolites, enabling a deeper understanding of how nanoparticles interact with molecular systems and influence plant responses to biotic and abiotic stresses (Muthamilarasan et al., 2019; Yang et al., 2021). This review critically summarizes recent advances in the application of SeNPs for enhancing plant tolerance to biotic and abiotic stresses, integrating insights from physiological, biochemical, and omics-based approaches.

## 2 Se in the plant-soil system

### 2.1 Sources and bioavailability of Se in soil

Se is a naturally occurring trace element whose distribution varies across the biosphere, being present in the lithosphere, hydrosphere, pedosphere, and atmospheric/open environments (Floor and Román-Ross, 2012; El-Ramady et al., 2016; White, 2016). The concentration of Se in soil is a primary determinant of its availability to plants, with soils considered Se-deficient when they contain < 0.5 mg kg^-^¹, and Se-enriched or seleniferous when concentrations exceed 4.0 mg kg^-^¹. On average, Se occurs at approximately 5 × 10^-^² mg kg^-^¹ in the Earth’s crust and 2 × 10^-4^ mg L^-^¹ in seawater (Fordyce, 2013; Huang et al., 2023; Moulick et al., 2024). However, Se bioavailability is influenced not only by its total concentration; it also depends on its chemical speciation, with Se occurring in soils primarily in various organic forms (e.g., selenomethionine derived from decomposed plant residues) as well as inorganic forms, including elemental Se (Se^0^), selenide (Se²^-^), selenite (SeO_3_²^-^), and selenate (SeO_4_²^-^).

Soil pH is a key factor influencing Se availability, with neutral to alkaline conditions generally enhancing Se bioavailability (Fordyce, 2013; Moulick et al., 2024). Thermodynamic (redox) conditions further determine the dominant Se species in soil. Under highly oxidized conditions (pe + pH > 15**),** Se predominantly exists as selenate (SeO_4_²^-^), which is highly soluble and readily available for plant uptake. In moderately oxidized, acidic-to-neutral soils (7.5 < pe + pH < 15), selenite (SeO_3_²^-^) becomes dominant and is more strongly adsorbed onto iron and aluminum oxides, thereby reducing its mobility (Séby et al., 2001; White, 2016; Hu et al., 2023). In contrast, under reducing, acidic, and organic matter–rich conditions, elemental Se, selenides, and selenium sulfide compounds prevail, exhibiting low solubility and limited plant availability (Tolu et al., 2022; Fu et al., 2025). In comparison, SeNPs exhibit relatively higher solubility and mobility under similar conditions, which may enhance their bioavailability (Tolu et al., 2022; Khan et al., 2023; Fu et al., 2025). Additionally, soil calcium carbonate plays an important role in regulating Se availability by buffering soil pH toward neutral to slightly alkaline conditions, thereby promoting the formation and mobility of selenate, the most phytoavailable form of Se for uptake via sulfate transporters (Tian et al., 2016; Moulick et al., 2024). Consequently, calcareous soils often exhibit greater Se availability and plant uptake compared with acidic soils (Tian et al., 2016; Moulick et al., 2024). SeNPs can be applied through soil amendment or foliar priming techniques. Due to their nanoscale size and high surface area, SeNPs can dissolve and transform into bioavailable Se forms in soil. They are primarily absorbed through root hairs and root tips, entering root cells via active or passive transport mechanisms and subsequently translocated throughout the plant. In contrast, foliar application enables SeNPs to enter leaves through stomata, followed by systemic transport to other plant parts. Foliar priming is often considered more efficient, as it provides rapid delivery, higher bioavailability, and improved assimilation compared with soil application (Qin et al., 2025).

### 2.2 Uptake, transport, and metabolism in plants

**Figure 1:**
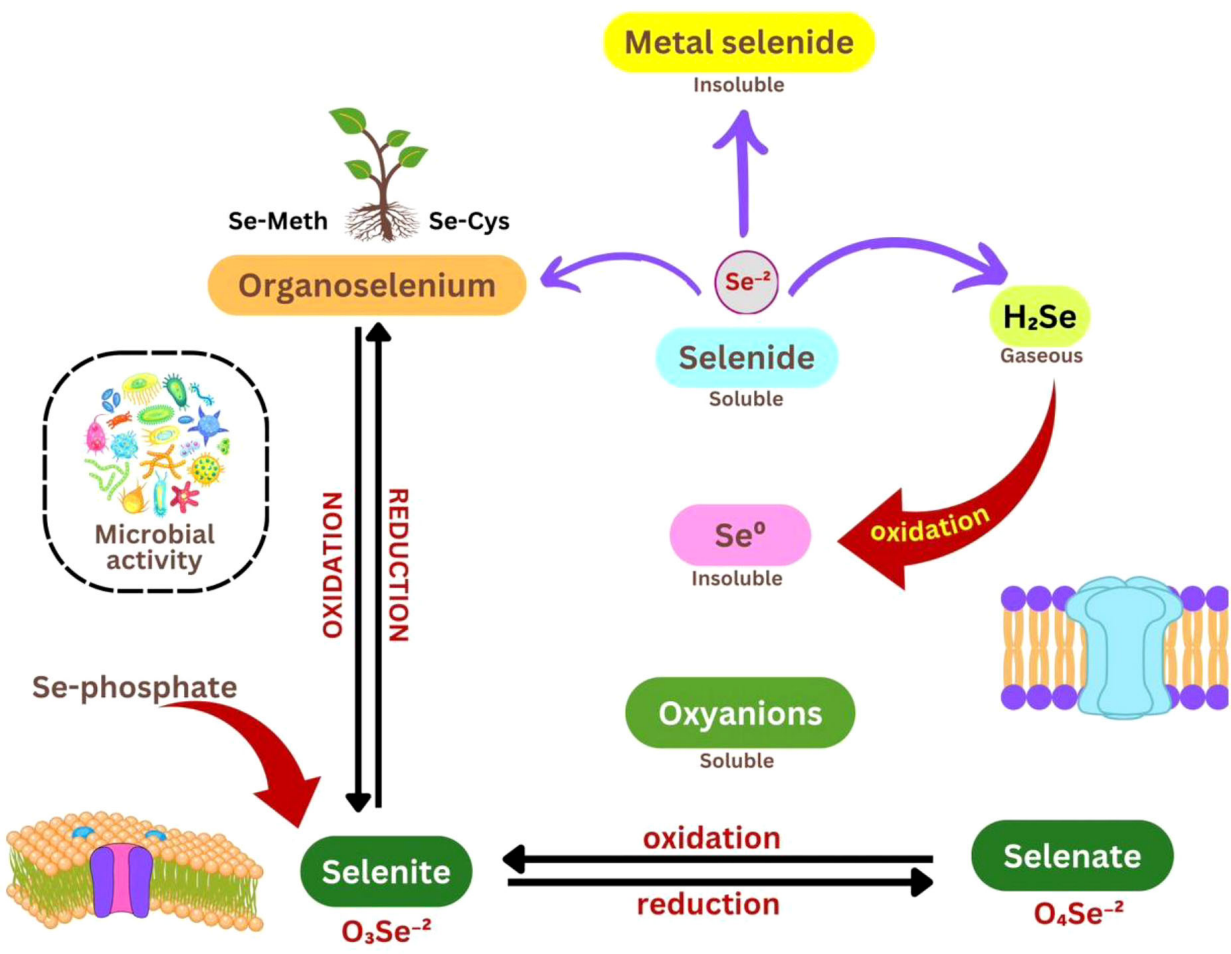
Conceptual overview of Se chemical forms, redox transformations, and microbial interactions in the soil–plant environment. Se occurs as soluble oxyanions, including selenate (SeO_4_²^-^) and selenite (SeO_3_²^-^), which undergo oxidation–reduction processes. Selenite can be converted into organoselenium compounds (e.g., Se-methionine and Se-cysteine) or reduced to elemental Se (Se^0^) and selenide (Se²^-^). Selenide may form insoluble metal selenides or volatilize as hydrogen selenide (H_2_Se). Microbial activity regulates these transformations, influencing Se bioavailability, mobility, and plant uptake.

Plants absorb selenium (Se) from the soil predominantly as selenite (SeO_3_²^-^), selenate (SeO_4_²^-^), and organic Se compounds (**Figure 1**) (Chao et al., 2022; Chen et al., 2025). For selenate uptake, plants utilize sulfate transport pathways due to the structural similarity between SeO_4_²^-^ and SO_4_²^-^. Two SULTR2 isoforms, *SULTR2;1* and *SULTR2;2*, facilitate the transport of selenate into the vascular system (El Mehdawi et al., 2018; Khan et al., 2022). In addition, high-affinity sulfate transporters *AtSULTR1;1* and *AtSULTR1;2* is primarily responsible for Se (VI) uptake, whereas *AtSULTR2;1*, *AtSULTR2;2*, and *AtSULTR3;5* mediate long-distance translocation from roots to shoots (El Mehdawi et al., 2018; Takahashi, 2019). Selenite uptake occurs mainly via phosphate transporters and aquaporins, although *SULTR1;2* has also been implicated under certain conditions (Xu et al., 2023; Lei et al., 2025).

Organic forms of Se are also efficiently absorbed, as amino acid permeases facilitate the transport of Se-containing amino acids such as selenomethionine (SeMet) and selenocysteine (SeCys), often at higher rates than inorganic Se forms in some plant species (Wang et al., 2022). Following uptake, Se is primarily metabolized in the plastids and cytosol of leaf cells (Schiavon and Pilon-Smits, 2017). Once translocated to leaves, selenate is activated by ATP sulfurylase to form adenosine 5′-phosphoselenate (APSe), which is subsequently reduced by adenosine 5′-phosphoselenate reductase to selenite (Schiavon et al., 2015; Xiao et al., 2025). Selenite is then further reduced to selenide via the sulfite reductase pathway, following a route analogous to sulfur assimilation (Bekturova et al., 2021). Selenide is incorporated into selenocysteine (SeCys) through the cysteine synthase complex (White, 2018). A portion of SeCys is further converted to selenocystathionine by cystathionine-γ-synthase and subsequently to selenohomocysteine by cystathionine-β-lyase, which serves as a precursor for selenometionine (SeMet) biosynthesis via methionine synthase (Cheng et al., 2025; Du et al., 2025).

Plants also employ detoxification mechanisms to prevent the misincorporation of Se-containing amino acids into proteins. S-adenosyl-L-methionine: methionine S-methyltransferase and selenocysteine methyltransferase catalyze the conversion of SeMet and SeCys into their non-proteinogenic methylated forms, methylselenomethionine (MeSeMet) and methylselenocysteine (MeSeCys), respectively (White, 2018; Hu et al., 2022). These methylated forms can be further volatilized and released from plant tissues, thereby reducing internal Se accumulation (Freeman et al., 2010). This mechanistic framework of Se uptake and assimilation (**Figure 1**) provides a basis for understanding Se subsequent physiological roles, including its roles in enhancing antioxidant defense machinery and improving photosynthetic performance, which are discussed in later sections.

## 3 Se and nanoselenium in combating specific abiotic stresses

### 3.1 Metal and metalloid toxicity

Nanoparticles, owing to their minute size and high reactivity, can be effectively transported throughout plant systems. They play a key role in conferring tolerance to metal toxicity by enhancing yield, strengthening plant defense systems, promoting the production of protective compounds, and regulating gene expression associated with metal transport, thereby reducing the adverse effects of metal toxicity (El-Mogy et al., 2025; Yang et al., 2025). Se plays a vital role in reducing the phytotoxicity of heavy metals and metalloids like cadmium (Cd), arsenic (As), and chromium (Cr). A primary mechanism through which Se alleviates metal/metalloid toxicity is by restricting their uptake and root-to-shoot translocation. This confinement reduces the mobility of the toxic ions and limits their metabolic impact in aerial tissues (AlYemeni et al., 2018; Hasanuzzaman et al., 2021; Asgher et al., 2023; Tian et al., 2024). In Cd-stressed tomato plantlets, reduced transport of Cd to shoots and leaves, along with Se-mediated inhibition of root-to-shoot Cd translocation, has been reported (AlYemeni et al., 2018). This is further achieved by modulating subcellular distribution in roots, such as decreasing metal accumulation in plastids and mitochondria while increasing sequestration in vacuoles and ribosomes (Zhao et al., 2019). Furthermore, the shielding role of Se involves enhanced phytochelatin (PC) synthesis, formation of non-toxic Se–metal complexes, and restriction of metal translocation from roots to aerial tissues, thereby reducing metal toxicity (Hawrylak-Nowak et al., 2014).

Applications of Se (IV) and (VI) enhance photosynthetic pigment content and overall physiological performance under metal stress (Abdelsalam et al., 2025a). For instance, tomato plants cultivated under Cd stress showed increased photosynthesis and biomass following Se application (Su et al., 2022). The impact of Se-based seed priming in rice under As stress improved germination by 9% and increased root, shoot, and total biomass by 1.6-, 1.3-, and 1.4-fold, respectively, compared with untreated stressed plants (Moulick et al., 2016). Likewise, in a study on CuO nanoparticles synthesized from *Melia azedarach* (MA-CuONPs), nanoparticle application reduced Cd uptake by enhancing antioxidant activity and mitigating heavy metal toxicity in *Triticum aestivum* (Naz et al., 2025). A summary of the beneficial effects of exogenous Se on various species under metal/metalloid toxicity is provided in **Table 1**.

**Table 1:** Effects of exogenous Se and Se-NPs application on several species in the presence of metal/metalloid toxicity.

Plant species	Concentration	Metal/metalloid	Beneficial outcomes	Reference
*Brassica campestris*	0.1 mg L^-1^ Se	Cr (1 mg L^-1^)	Enhanced growth and reduced Cr uptake.	Zhao et al., 2019
*Brassica juncea*	Se (50 µM)	Cd;100 and 200mg L^-1^ CdSO_4_.8H_2_O	Enhanced the activities of SOD, GR, APX and reduced Cd uptake	Ahmad et al., 2016
*Brassica napus* and *Brassica juncea*	3 μM L^-1^ Se	Cd (50 μM L^-1^)	Improved chlorophyll content and antioxidant activity while reduced ROS under Cd stress	Zhang et al., 2020
*Cucumis sativus*	4–8 mg L^-1^ Se	Cd (20–25 mM)	Improved growth and agronomic attributes under Cd stress	Sun et al., 2016)
*Glycine max*	10 to 25 μM L^-1^	As (25 μM L^-1^)	Enhanced the expression of stress-responsive genes, transcription factors, and molecular chaperones and reduced ROS under As stress.	Zeeshan et al., 2023
*Oryza sativa*	Na_2_SeO_3_ (0, 5, 10 and 25 μM)	NaAsO_2_ (25 μM)	Improved growth and biochemical parameters under As stress	Chauhan et al., 2017
*Oryza sativa*	0.5-1.25 mg Se L^-1^	As (5.0 mg As L^-1^)	Reduced As translocation and enhanced the concentration of mineral nutrients.	Moulick et al., 2023
*Oryza sativa*	0.5-1.0 mg kg^-1^	As (25- 100 μM/kg)	Restraining the amount of carbohydrates and/or the absorption of nutrients.	Bhadwal and Sharma, 2022
*Raphanus sativus*	1, 3, 6, 12 and 24 mg Na_2_SeO_4_kg^-1^ soil	As; 30mg As (III) kg^-1^ soil	Rise in activity of DHAR, APX and GR under As stress	Hu et al., 2020
*Raphanus sativus*	2–8 mg L^-1^ Se	Cd (5–10 mg L^-1^)	Decreased MDA content, improved CAT, APX, GPX activities enhance antioxidant defense mechanism and tolerance and decreased Cd uptake, distribution etc.	Auobi Amirabad et al., 2020
*Raphanus sativus*	1–24 mg kg^-1^	As (30 mg kg^-1^)	Improved GR, DHAR as well as APX activities and minimized As accumulation.	Hu et al., 2021
*Raphanus sativus*	2,4 and 8 mgL^-1^ Na_2_SeO_3_	Cd; 5 and 10 mg L^-1^ CdSO_4_	Enhanced the activities of APX, CAT and GPX while MDA content was reduced.	Auobi Amirabad et al., 2020
*Solanum lycopersicum*	10 µM of Na_2_SeO_3_	Cd	GR, SOD, CAT and APX activities were enhanced	AlYemeni et al., 2018
*Solanum lycopersicum*	SeNPs0, 100, 300mg L^-1^	Cd	Improved the physiological and biochemical attributes and reduced the Cd accumulation.	Ahmad et al., 2024
*Vicia faba*	Na_2_SeO_3_ (1.5 or 6 μM)	Pb (NO_3_)_2_ (50 μM)	Chlorophyll level improved, while MDA, H_2_O_2_, and O_2_• buildup decreased	Mroczek-Zdyrska et al., 2017
*Triticum aestivum*	0.4 and 0.8mg Se^6+^ kg^-1^ soil	Pb; 50 and 100mg Pb^2+^ Kg^-1^ soil	There is rise in GPX and GR activity	Balakhnina and Nadezhkina, 2017
*Triticum aestivum*	CuO NPs	Cd concentration (30 ppm)	Increased growth and photosynthesis	Naz et al., 2025

### 3.2 Salinity stress

Salinity has become a major global constraint due to its severe abiotic stress effects that adversely impact plant growth and development by disrupting multiple metabolic processes (Atta et al., 2023). The beneficial role of Se and SeNPs under various levels of salt stress has been demonstrated in several studies. Exogenous application of Se and SeNPs enhances seed germination in *Tagetes patula*, *Brassica napus* (El-Badri et al., 2021, El-Badri et al., 2022a), *Brassica rapa* (Hussain et al., 2024), *Triticum aestivum* (Ghazi et al., 2022), *Brassica campestris* (Sarkar and Kalita, 2022b), *Lallemantia iberica*, *Cichorium intybus*, and *Alyssum homalocarpum* (Amerian and Khosravi, 2022), *Oryza sativa* (Adhikary et al., 2022), and *Sorghum bicolor* (Nie et al., 2023) by improving α-amylase activity, seed microstructure, and seed vigor under salt stress. Several studies have demonstrated the beneficial role of Se and SeNPs on plant growth and crop productivity. For example, application of Se significantly improved growth attributes in *Solanum lycopersicum* (Wu et al., 2023), *Brassica oleracea* (Kucukyumuk and Suarez, 2021), *Lippia citriodora* (Ghanbari et al., 2023), *Panicum miliaceum* (Rasool et al., 2023), *Fragaria × ananassa* (Pourebrahimi et al., 2023a), *Stevia rebaudiana* (Shahverdi et al., 2020), *Vigna unguiculata* (Manaf, 2016), *Mentha suaveolens* (Kiumarzi et al., 2022), and *Phaseolus vulgaris* (Admasie et al., 2023) under salt stress. Moreover, under salt stress, SeNPs significantly enhance growth performance in *Cucumis sativus* (Shalaby et al., 2021), *Momordica charantia* (Sheikhalipour et al., 2021a), *Gossypium barbadense* (Saad-Allah et al., 2022), *Oryza sativa* (Badawy et al., 2021), *Triticum aestivum* (Soliman et al., 2023), *Physalis alkekengi* (Abdi et al., 2023), and *Glycine max* (Wang et al., 2023a).

Multiple studies have demonstrated that Se and SeNPs improve mineral nutrition under salinity stress by reducing Na^+^ uptake and enhancing the accumulation of essential ions such as K^+^, Mg²^+^, and Ca²^+^. Using sodium selenate, sodium selenite, and SeNP treatments, positive effects were reported in maize (Jiang et al., 2017; Xu et al., 2021; Khalil et al., 2022), garlic (Astaneh et al., 2018), strawberry (Soleymanzadeh et al., 2020), bitter melon (Sheikhalipour et al., 2023), pansy (Javadi et al., 2020), grapevine (Aazami et al., 2022), sunflower (Habibi, 2017b), maize (Wang et al., 2023b), and *Vigna radiata* (Alrashidi, 2022). Se and SeNPs alleviate salinity-induced damage to the photosynthetic machinery, improving photosynthetic pigments, gas exchange parameters, and overall photosynthetic **e**fficiency in strawberry (Pourebrahimi et al., 2023b), snap bean (Farag et al., 2022), olive (Regni et al., 2021), maize (Khalil et al., 2022), tomato (Morales-Espinoza et al., 2019), parsley (Habibi, 2017a), stevia (Sheikhalipour et al., 2021b), and *Vigna radiata* (Alrashidi, 2022). By reducing electrolyte leakage, improving relative water content, and enhancing membrane stability index, Se and SeNPs strengthen water relations and membrane integrity under salinity stress in tomato (Hajlaoui et al., 2023), sorrel (Kong et al., 2005), onion (Semida et al., 2021), olive (Regni et al., 2021), squash (Alsamadany et al., 2023), beans (Rady et al., 2021), and wheat (Desoky et al., 2021).

Se ameliorates salt stress through several key mechanisms. A critical mechanism is the maintenance of ionic (K^+^/Na^+^) homeostasis (Mishra et al., 2025; Ashgehsou et al., 2026). Salinity typically leads to excessive Na^+^ accumulation and a decline in K^+^ levels, thereby reducing the K^+^/Na^+^ ratio (Assaha et al., 2017). Se helps maintain this ratio by limiting Na^+^ accumulation and promoting K^+^ uptake, thus mitigating ionic toxicity (Hawrylak-Nowak, 2009; Rasool et al., 2022). For example, treatment with Se significantly increased shoot K^+^ concentration in *Zea mays* (Jiang et al., 2017) and *Osteospermum ecklonis* (Ashgehsou et al., 2026) under salt stress. Se also supports the upregulation of NHX1, a key vacuolar Na^+^/H^+^ antiporter responsible for Na^+^ sequestration into vacuoles (El-Badri et al., 2022b). In addition, Se promotes osmotic adjustment by modulating osmoprotectants under salt stress. In cucumber leaves, Se treatments significantly increased proline accumulation (Hawrylak-Nowak, 2009). Se regulates proline metabolism by enhancing glutamate kinase activity (proline synthesis) and reducing proline oxidase activity (proline degradation) in wheat (Elkelish et al., 2019). Furthermore, Se protects the photosynthetic apparatus by improving photosynthetic efficiency and preserving chloroplast ultrastructure under salinity stress (Jiang et al., 2017). The beneficial outcomes of Se application for various plant species under salinity stress are summarized in **Table 2**.

**Table 2:** Effect of exogenous Se and Se-NPs on plants under salinity stress.

Plant species	Se form	Se concentration	Beneficial outcomes	References
*Allium sativum*	Na_2_SeO_4_	4, 8 or 16 mg L^-1^	Improved photosynthetic pigments, phenolic content, activities of antioxidant enzymes and reduced the levels of MDA & electrolyte leakage.	Astaneh et al., 2019
*Cucurbita pepo*	Na_2_SeO_4_	15 g Se per hectare	Improved plant growth, yield and quality attributes and also enhanced gene expression, photosynthetic efficiency and antioxidant defense mechanisms under salt stress	Alsamadany et al., 2023
*Helianthus annuus*	Na_2_SeO_4_	5 mg kg^-1^ Se	Improved growth and physiological parameters by blocking Na translocation and amelioration.	Habibi, 2017a
*Melissa officinalis*	Se-NPs	50 or 100 mg L^-1^	Improved growth and antioxidant mechanisms while reducing lipid peroxidation.	Ghasemian et al., 2021
*Olea europaea*	Na_2_SeO_4_	10 or 30 mg L^-1^	Improved growth and photosynthesis and facilitated the restoration of ionic homeostasis under salt stress.	Regni et al., 2021
*Panicummiliaceum*	Na_2_SeO_4_	1-10 µM	Improved salt tolerance by increasing the concentration of osmoprotectants.	Rasool et al., 2023
*Setaria italicand Panicummiliaceum*	Na_2_SeO_3_	1 Mμ	Enhanced the activities of antioxidant enzymes, osmolytes, and decreased the levels of ROS.	Shah et al., 2020
*Solanum lycopersicum*	Se-NPs	1–20 mg L− 1	Improved the growth and various physiological and biochemical attributes under stress.	Morales-Espinoza et al., 2019
*Triticum aestivum*	Selenium chloride	2,4 or 8 μm	Improved the physiological and biochemical parameters by reducing Na accumulation.	Desoky et al., 2021
*Vitis vinifera*	Na_2_SeO_4_	5 or 10 mg L^-1^	Reduced ROS and electrolyte leakage and improved growth and photosynthetic efficiency and antioxidant mechanisms.	Karimi et al., 2020
*Zea mays*	Na_2_SeO_3_	1, 5 or 25 μm	Promoted plant growth and development by enhancing photosynthesis, impairments in chloroplast ultrastructure and activities antioxidant enzymes, and also promoted K^+^ Na^+^ balance	Jiang et al., 2017

### 3.3 Drought stress

Water deficit leads to drought stress, which reduces shoot and young branch growth and photosynthetic pigment content while often promoting root growth (Seleiman et al., 2021; Peer et al., 2025). Se-application mitigates drought stress by modulating antioxidant defense systems and maintaining osmotic balance. For instance, in studies on cucumber and *Trifolium repens*, it was reported that the capacity of these crops growing under drought and pre-treated with Se to generate ROS (O_2_^-^, H_2_O_2_, and OH^-^) was significantly lower compared with plants exposed to drought alone (Wang, 2011; Jóźwiak and Politycka, 2019). This reduction in oxidative stress was evidenced by decreased MDA content and lower damage index values. The exogenous application of Se in rice enhanced drought tolerance by increasing total antioxidant capacity, particularly in shoots, through elevated activities of CAT, APX, GPX, and SOD, accompanied by reduced MDA and H_2_O_2_ levels (Kumar et al., 2014). Se also improves plant water status and osmotic adjustment. For example, in *Camelina sativa*, a significant increase in chlorophyll content as well as CAT, POX, APX, and SOD activities was observed following Se priming under drought conditions (Ahmad et al., 2021). In *Cucumis sativus*, Se application enhanced relative water content, improved ROS scavenging, and strengthened antioxidant defense mechanisms (Jóźwiak and Politycka, 2019; Tsivileva, 2025). Drought-induced alterations in plant–water relations typically lead to stomatal closure and reduced photosynthetic activity; however, Se helps mitigate these adverse effects (Jiang et al., 2017; Liu et al., 2022). The specific effects of Se on various species under drought conditions are detailed in **Table 3**.

**Table 3:** Effect of exogenous selenium and Se-NPs against drought stress.

Plant species	Se Form	Se concentration	Beneficial outcomes	References
*Brassica napus*	Na_2_SeO_3_	21 g h^-1^	Increase the activities of defense enzymes	Pazoki et al., 2010
*Camelina sativa*	Na_2_SeO_3_	5.1 mg L^-1^	Substantial rise in chlorophyll content as well as CAT, POX, APX, and SOD activity.	Ahmad et al., 2021
*Cucumis sativus*	Na_2_SeO_3_	1–10 μM	Increased percentage of water enhances ROS scavenging and antioxidant defense mechanism.	Jóźwiak and Politycka, 2019
*Olea europaea*	Na_2_SeO_3_	50 and 150 mg Se L^-1^	Increase CAT, APX, and GPOX activity	Proietti et al., 2013
*Oryza sativa*	Na_2_SeO_3_	0.5–2.0 mg kg^-1^	Stimulated the antioxidant defense mechanisms and improved photosynthesis.	Andrade et al., 2018
*Oryza sativa*	Na_2_SeO_3_	0–20 mg L^-1^	Improved proline, relative water content, and membrane stability index.	Patnaik et al., 2023
Soybean	Se-NPs	100–200 mgL^-1^	Improved drought tolerance by boosting antioxidant defense mechanisms and photosynthetic pigments.	Zeeshan et al., 2023
Tomato	Se-NPs	0 and 4mgL^-1^	Improved drought tolerance by boosting stress-related metabolites and antioxidant defense mechanisms also upregulating CRITISO &bZIP, increasing miR-172 under normal watering, but reducing under drought stress.	Neysanian et al., 2020
*Triticum aestivum*	Na_2_SeO_4_	25 µM	Improved antioxidative defense mechanisms and facilitated the quick removal of ROS	Hasanuzzaman et al., 2024
*Triticum aestivum*. cv. Giza	Na_2_SeO_4_	10 and 20 mg Se L^-1^	Increase CAT, SOD as well as AsA and GSH activity, down regulate POD and decrease α-TQ and proline content.	Ibrahim, 2014
Wheat	Na_2_SeO_3_	40 mg Se L^-1^	Increase CAT, POX and APX activity	Nawaz et al., 2015
*Zea mays*	Na_2_SeO_4_	40 mg Se L^-1^	Increase SOD, CAT, POX, and APX activity, Increase leaf chlorophyll a, b and carotenoid contents.	Nawaz et al., 2016
Potato	Se-NPs	50 mg L^-1^	Improved drought resistance through physiological and transcriptomic modulation.	Al-Amri, 2026

### 3.4 Temperature stress (high and low)

Extreme temperature stress results in leaf senescence, which inhibits photosynthesis, disrupts membrane integrity, and leads to chlorophyll degradation, while also enhancing the production of ROS under heat stress (Peer et al., 2020; Lunn et al., 2022; Upadhyay et al., 2022). Se protects plants from temperature extremes by activating antioxidant defense systems and stabilizing cellular structures. Under high-temperature stress in wheat, Se application increased phenolic and chlorophyll contents while reducing H_2_O_2_ and MDA levels (Iqbal et al., 2015). In *B. napus*, levels of antioxidants such as GSH and AsA were enhanced, and the activities of GPX, CAT, GR, DHAR, MDHAR, and glyoxalase enzymes were significantly upregulated following Se application (Hasanuzzaman et al., 2014). Se also promotes the synthesis of osmoprotectants under heat stress; in cucumber, glycine betaine, proline, and total soluble sugars were significantly increased following Se treatment (Balal et al., 2016). In *Chrysanthemum* under high temperature, Se application improved plant longevity and increased flower production by mitigating the adverse effects of heat stress and activating antioxidant defense mechanisms (Seliem et al., 2020). Similarly, under low-temperature stress, Se enhances physiological performance and reduces oxidative damage, as observed in strawberry seedlings (Huang et al., 2018). The protective effects of Se against extreme temperature stress across different plant species are summarized in **Table 4**.

**Table 4:** Effect of exogenous selenium under conditions of extreme temperature.

Species name	Concentration	Beneficial outcomes	References
*Brassica napus*	25 µM Na_2_SeO_4_	Improved growth and tolerance by stimulating antioxidant defense mechanisms	Hasanuzzaman et al., 2014
*Chrysanthemum morifolium Ramat*	50-200mg L^-1^	Increased plant longevity and the quantity of flowers blooming. Overcome negative effects of temperature stress. Activation of antioxidant defense mechanism.	Seliem et al., 2020
*Cucumis sativus*	8 µM Na_2_SeO_4_	Improved growth and agronomic attributes by activating antioxidant defense mechanisms	Balal et al., 2016
*Gossypium hirsutum*	50–150 mg L^-1^ Se	A strong correlation exists between cotton seeds output, phenological and quality attributes upon Se supplementation.	Saleem et al., 2021
*Valerianella locusta*	50 mg Se L^-1^ as Na_2_SeO_4_	Enhanced plant growth as well as development and reduced oxidative stress due to increased guaiacol peroxidase (GPOX) and catalase activity, elevated levels of GSH, and decreased H_2_O_2_ buildup.	Hawrylak-Nowak et al., 2018
Wheat	2 and 4 mg Se L ^−1^ Na_2_SeO_4_	Glyoxalase I (Gly I) and glyoxalase II (Gly II); higher chloride concentration, accelerated growth, lowered H_2_O_2_ and MDA content, increased CAT and APX activity, increased phenolic contents, and increased chlorophyll contents.	Iqbal et al., 2015
*Zea mays*	5–15 μM Se	Enhanced the antioxidant enzyme mechanism as well as the scavenging of ROS.	Yildiztugay et al., 2017

## 4 General mechanisms of Se-mediated abiotic stress tolerance

When exposed to abiotic stresses, plants experience an overproduction of ROS, leading to oxidative damage. Se mitigates this damage by enhancing the plant’s antioxidant defense system. The reduction in ROS levels, mediated by the activation of antioxidant enzymes such as GPX, APX, and others, is a key factor contributing to the improved performance of Se-treated plants (Feng et al., 2013; Hasanuzzaman et al., 2024; Saeed et al., 2025). By enhancing antioxidant capacity, Se positively influences plant growth and stress tolerance (Zhu et al., 2017; Pereira et al., 2018; Saeed et al., 2025).

### 4.1 Enhancement of the antioxidant defense system

Se plays a significant role in the upregulation of numerous antioxidative enzymes, including CAT, POD, SOD, APX, and GPX. The application of Se to stressed plants reduces the accumulation of ROS, thereby protecting plants from oxidative damage (Hasanuzzaman et al., 2021; Liu et al., 2022; Hasanuzzaman et al., 2024). For instance, in rice under As toxicity, Se application increased the activities of antioxidant enzymes, including SOD, APX, and POD (Singh et al., 2018). Similarly, Se application enhanced antioxidant enzyme activity in cucumber under heat stress (Balal et al., 2016). This enhancement is not limited to enzymatic components; Se also promotes the synthesis of non-enzymatic antioxidants such as glutathione in *Arabidopsis* (Khan et al., 2022). In *Brassica napus* under high-temperature stress, levels of antioxidants (GSH and AsA) increased, along with elevated activities of GPX, CAT, GR, DHAR, MDHAR, and glyoxalase enzymes (Hasanuzzaman et al., 2014, Hasanuzzaman et al., 2018).

Se plays a crucial role in maintaining cellular redox homeostasis under stressful conditions through the enhancement of both enzymatic and non-enzymatic antioxidant systems. One of the primary functions of Se is to reduce excessive ROS production under stress conditions (Hasanuzzaman et al., 2020a). Se supplementation limits ROS overproduction by strengthening antioxidant defense mechanisms (Hasanuzzaman et al., 2020a, Hasanuzzaman et al., 2021; Saeed et al., 2025). This is evidenced by a consistent decrease in specific ROS; for instance, SeNP supplementation significantly enhances antioxidant enzyme activities such as SOD, CAT, PPO, and POX in carrot plants under drought stress (El-Batal et al., 2023). Likewise, Morales-Espinoza et al. (2019) demonstrated a marked increase in both enzymatic and non-enzymatic antioxidants, including APX, CAT, β-carotene, lycopene, and phenolic compounds, in tomato plants treated with SeNPs under salt stress. In *Trifolium repens* under drought, the capacity to produce O_2_^-^, H_2_O_2_, and OH^-^ was significantly reduced in Se-pretreated plants (Wang, 2011). This reduction in ROS directly translates to lower cellular damage, particularly reduced lipid peroxidation, as indicated by decreased malondialdehyde (MDA) levels. In wheat under heat stress, Se application reduced H_2_O_2_ and MDA contents (Iqbal et al., 2015). Similar effects were observed in *Vicia faba* under lead stress, where Se decreased the accumulation of MDA, H_2_O_2_, and O_2_•^-^ (Mroczek-Zdyrska and Wójcik, 2012; Mroczek-Zdyrska et al., 2017). Therefore, Se-mediated regulation of ROS scavenging and antioxidant enzymes represents a key mechanism for maintaining redox balance and protecting plant cells under stress conditions (Cartes et al., 2010).

### 4.2 Improvement of photosynthetic efficiency and membrane stability

Se plays a crucial role in protecting the photosynthetic apparatus from stress-induced damage. In drought-stressed crops such as rice and canola, Se supplementation has been shown to increase chlorophyll content and overall photosynthetic capacity (Andrade et al., 2018; Hemmati et al., 2019). This protective role extends to the physical structures involved in photosynthesis; Se has been found to stabilize thylakoid membranes and chloroplast stroma, thereby protecting photosynthetic structures under UV-induced stress (Liu et al., 2022; Moulick et al., 2024). By improving stomatal conductance and photosynthetic efficiency, Se supports carbon assimilation under adverse environmental conditions (Zhang et al., 2023a, Zhang et al., 2025a). The protection of cellular membranes is a critical part of this process, as Se maintains membrane integrity and reduces electrolyte leakage, ensuring the stability of chloroplasts and other organelles (Ghanbari et al., 2023; Saeed et al., 2025).

### 4.3 Modulation of osmoprotectants and secondary metabolism

Furthermore, by promoting the synthesis of non-enzymatic antioxidants such as GSH, osmoprotectants, and stress-responsive gene regulation, Se aids plants in mitigating stress by modulating intracellular osmolyte levels (Liu et al., 2022; Bandehagh et al., 2023). For instance, modulation of proline and GSH metabolism reduced Cd-induced oxidative stress in *Triticum aestivum* (Khan et al., 2015). In cucumber under heat stress, Se application increased GB, proline, and total soluble sugars (Balal et al., 2016). Se also modulates proline accumulation by enhancing glutamate kinase activity (proline synthesis) and reducing proline oxidase activity (proline degradation) (Elkelish et al., 2019). Under UV stress, Se stimulates the production of antioxidant enzymes and UV-absorbing compounds (Golob et al., 2018). The induction of these diverse compounds represents a comprehensive strategy through which Se enhances plant stress tolerance and resilience.

## 5 The nano-dimension: SeNPs

### 5.1 Synthesis of SeNPs

**Figure 2:**
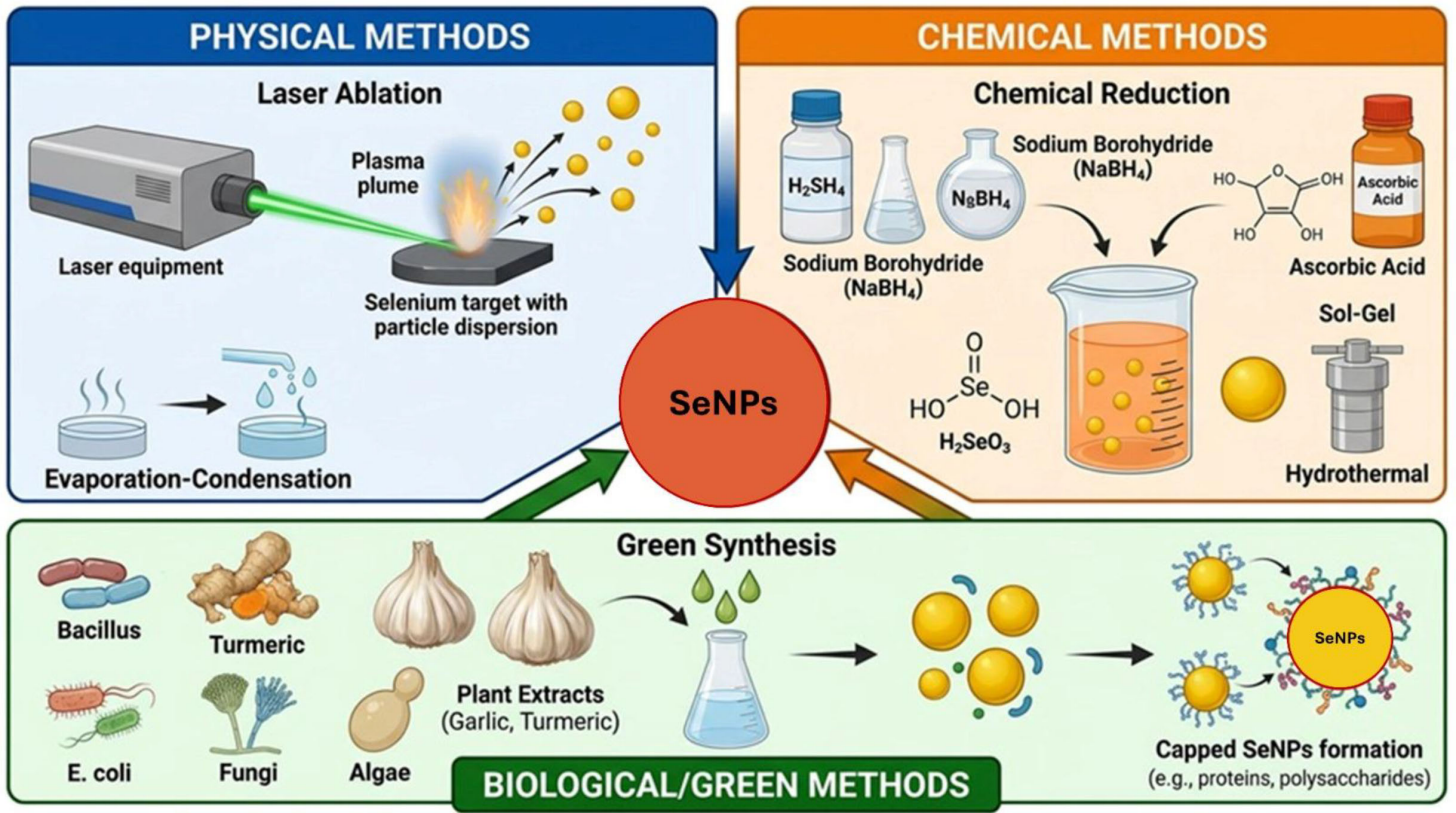
Schematic representation of SeNPs synthesis methods, including physical and chemical approaches, as well as biological synthesis.

The synthesis of SeNPs has evolved through three primary methodological approaches: physical, chemical, and biological techniques (**Figure 2**). Each approach offers unique advantages and faces specific limitations that influence their practical applicability in research and industrial settings (Geng et al., 2025).

Physical synthesis techniques include pulsed laser ablation, vapor deposition, and hydrothermal processes (Pyrzynska and Sentkowska, 2021). Pulsed laser ablation employs high-intensity lasers (e.g., a 532-nm laser with 10-ns pulses) to produce SeNPs with controlled sizes ranging from 3 to 18 nm (Sarkar et al., 2015). These methods offer advantages, including high surface purity, reduced contamination, and easy nanoparticle collection (Lin et al., 2021). However, physical approaches face significant limitations, including high energy consumption, stringent operational conditions, and elevated costs that restrict their widespread application (Dhawan et al., 2021). The requirement for specialized equipment and high thermal conditions further limits their scalability for large-scale production (Gunti et al., 2019).

Chemical synthesis involves the reduction of Se precursors using various reducing agents, including ascorbic acid, glutathione, hydrazine, and organic acids, often combined with stabilizing agents such as polyvinyl alcohol or chitosan (Sarkar et al., 2015; Zhang et al., 2023a). These methods provide excellent control over particle size and morphology, producing SeNPs with high activity and stability (Zhang et al., 2023a). Chemical approaches are relatively low-cost and enable the production of functional nanoparticles with controllable dimensions (Lin et al., 2021). Nevertheless, major limitations include the use of toxic chemicals, generation of hazardous waste, environmental pollution concerns, and the necessity for extensive purification steps (Ghareeb et al., 2025; Dhawan et al., 2021). The multistep synthesis process and potential toxicity of chemical residues also compromise biocompatibility for biomedical applications (Pyrzynska and Sentkowska, 2021).

Biological or “green” synthesis has emerged as the most promising approach, utilizing microorganisms (bacteria, fungi, algae) and plant extracts as reducing and stabilizing agents (Mikhailova, 2023). Bacterial species such as *Ralstonia eutropha*, *Klebsiella pneumoniae*, and *Bacillus cereus*, along with plant extracts from *Vitis vinifera*, *Emblica officinalis*, and mulberry leaves, have demonstrated effective SeNP production (Sarkar et al., 2015; Gunti et al., 2019; Zhang et al., 2023a). This approach offers multiple advantages: environmental friendliness, use of non-toxic solvents, operation at moderate temperatures, cost-effectiveness, and superior biocompatibility (Sampath et al., 2024; Pyrzynska and Sentkowska, 2021). Biogenic SeNPs exhibit enhanced stability due to natural biomolecule coatings that prevent aggregation and demonstrate reduced toxicity compared to chemically synthesized counterparts (Ghareeb et al., 2025; Singh et al., 2022). The single-step conversion process eliminates the need for additional stabilizers and reduces purification requirements (Dhawan et al., 2021). However, biological synthesis faces challenges, including reproducibility issues due to metabolite variations, potential difficulties in standardization, and sometimes lower stability requiring additional stabilizers (Ghareeb et al., 2025; Zhang et al., 2023b). The synthesis process can be time-consuming, with some methods requiring up to one week for complete reduction (Deepa and Ganesan, 2015).

The three fundamental approaches to SeNPs each serve specific needs within the research and application landscape. While physical and chemical methods provide precise control over particle characteristics, biological synthesis has emerged as the preferred approach for biomedical applications due to its environmental sustainability, cost-effectiveness, and enhanced biocompatibility.

### 5.2 Unique advantages of SeNPs over bulk selenium

In comparison to its organic and inorganic Se counterparts, such as selenite (SeO_3_²^-^) and selenate (SeO_4_²^-^), SeNPs have attracted the attention of numerous researchers because of their lower toxicity, enhanced biocompatibility, and greater bioavailability (Ran et al., 2024b). Their high surface area-to-volume ratio increases their reactivity, allowing them to interact more effectively with biological systems and regulate antioxidant enzyme activities (Wahab et al., 2024). SeNPs exhibit superior activity compared with bulk materials due to their smaller size and improved surface area, as well as their unique physicochemical properties, stability, and morphology (Ingle et al., 2014). This enables them to function as efficient micronutrient sources, enhancing plant performance and improving tolerance to environmental stresses more effectively than conventional Se forms.

### 5.3 Impact of SeNPs on plant growth, physiology, and metabolism

Depending on plant species and the concentration of application, SeNPs have significant effects on plant metabolism. They act as catalysts and enhance the plant antioxidant defense system, thereby improving their capacity to tolerate biotic and abiotic stresses (Zohra et al., 2021). For instance, under elevated salt stress conditions, which increase MDA and H_2_O_2_ levels, growth and photosynthetic pigment content in *Zea mays* were enhanced following Se application (Xu et al., 2021). Applications of green-synthesized SeNPs increased the activity of SOD, CAT, and other antioxidant enzymes, thereby improving the resistance of maize and strawberry plants to abiotic stress conditions (Zahedi et al., 2019; Wang et al., 2023b). Furthermore, SeNPs are effective elicitors of secondary metabolism, as studies have shown that nano-selenium application stimulates the production of secondary metabolites in plants (Ghanbari et al., 2023; Mazhar et al., 2024). For example, foliar application of SeNPs increased total flavonoid content, total phenolic compounds, and vitamin C levels in celery (Li et al., 2020). In salt-stressed wheat plants, SeNPs significantly increased the accumulation of phenols, osmolytes, and flavonoid compounds, including proline, sugars, and glycine betaine (Elkelish et al., 2019; Hasanuzzaman et al., 2024; Zafar et al., 2024). The enhanced accumulation of phenolics and flavonoids strengthens the antioxidant defense system, thereby minimizing ROS-induced damage to cellular organelles (Samynathan et al., 2023).

### 5.4 SeNPs in biofortification and stress alleviation

SeNP-based biofortification is increasingly important as it enhances plant stress tolerance and metabolic efficiency. To combat Se deficiency, numerous plant species have been biofortified with Se using its nanoparticles (Schiavon et al., 2020; Garza-García et al., 2023; Escalona-Tenorio et al., 2024). SeNPs are increasingly applied in agriculture through methods such as hydroponics, foliar spraying, and soil or root-priming techniques. SeNPs have recently emerged as an effective alternative to conventional Se salts such as selenate and selenite for crop biofortification and stress mitigation. Compared with ionic Se forms, SeNPs often exhibit greater stability, reduced toxicity, and controlled Se release, which can improve Se uptake efficiency while minimizing the risk of phytotoxicity and environmental losses (Hasanuzzaman et al., 2020a; Garza-García et al., 2023). Several studies have demonstrated that SeNPs can perform equally well or even outperform conventional Se sources in enhancing plant growth under various stresses, including biotic (Joshi et al., 2019; Amin et al., 2021), cold (Sayed et al., 2024), heat (Shalaby et al., 2021), drought (Zeeshan et al., 2024), salinity (Rady et al., 2021), and heavy metal stress (Qin et al., 2025). Wang et al. (2021) reported that nano-Se application in rice grown in Pb- and Cd-contaminated soils significantly improved plant growth, photosynthetic performance, chlorophyll content, and the expression of stress-responsive proteins compared with untreated plants. Similarly, SeNP-based biofortification strategies have been applied in hydroponic, foliar, and soil systems to enhance Se accumulation in edible plant tissues while improving crop nutritional quality (Lara et al., 2019; Schiavon et al., 2020; Escalona-Tenorio et al., 2024). Beyond increasing Se content, SeNPs provide additional physiological benefits by strengthening antioxidant defense systems, regulating ROS homeostasis, and improving membrane stability and metabolic functions under stress conditions.

These mechanisms enable plants to better withstand environmental stresses such as heavy metal toxicity, salinity, and drought (Hasanuzzaman et al., 2020a). Owing to their unique physicochemical properties and multifunctional roles, SeNPs are increasingly considered a promising strategy for integrated crop biofortification, stress mitigation, and enhancement of nutritional quality within sustainable agricultural systems (Garza-García et al., 2023; Burmistrov et al., 2025).

## 6 Role of Se in enhancing tolerance against biotic stresses

In recent years, research has highlighted the importance of Se in mediating plant responses to biotic stresses such as pests and pathogens, which are major limiting factors in agricultural productivity. The following sections provide a comprehensive overview of the role of Se in enhancing plant tolerance to various biotic stresses.

### 6.1 Se-mediated defense against plant pathogens

**Figure 3:**
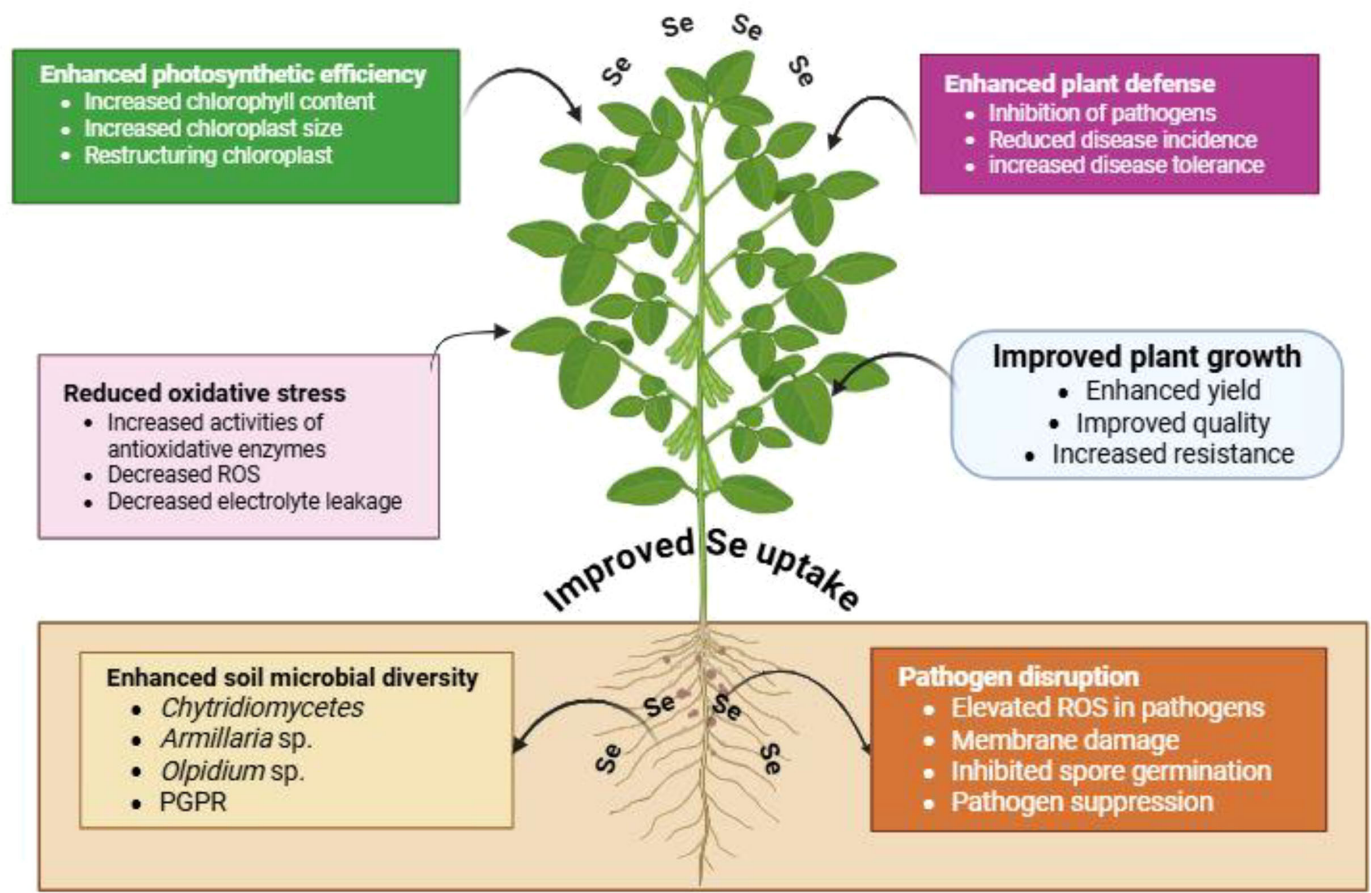
Se-mediated regulation of plant growth, defense, and stress tolerance. Se enhances photosynthetic efficiency by increasing chlorophyll content and improving chloroplast structure. It alleviates oxidative stress through activation of antioxidant enzymes, reducing reactive oxygen species (ROS) and electrolyte leakage. Se strengthens plant defense by inhibiting pathogens and reducing disease incidence. It also improves soil microbial diversity, including plant growth-promoting rhizobacteria (PGPR). Additionally, Se disrupts pathogen activity via ROS-induced damage and inhibition of spore germination, resulting in improved growth, yield, quality, and stress resilience.

Se enhances plant resistance to pathogens by inducing structural and functional changes in soil microbial communities, thereby preventing pathogen invasion (Li et al., 2023). It has been shown that healthy plants harbor greater microbial diversity in the rhizosphere compared with diseased plants, along with higher soil Se content (≥ 0.4 mg kg^-^¹). Increased microbial diversity and the relative abundance of PGPR enhance Se bioaccumulation in plant tissues, which strengthens plant defense against pathogen invasion and reduces the incidence of soil-borne diseases (Liu et al., 2019). Se improves the diversity of beneficial microorganisms while reducing the relative abundance of pathogenic microbes, such as *Chytridiomycetes*, *Microbotrymycetes*, *Coniosporium* sp., *Armillaria* sp., and *Olpidium* sp. in soil (Liu et al., 2019). Various studies have demonstrated the inhibitory effects of Se on phytopathogens such as *Pityrosporum ovale* (Brotherton, 1967), *Alternaria tenuis*, *Aspergillus funiculosus* (Razak et al., 1991), *Penicillium expansum* (Wu et al., 2014), *Botrytis cinerea* (Wu et al., 2015), *Sclerospora graminicola* (Nandini et al., 2017), *Fusarium* spp. (Kornaś et al., 2019), *Pectobacterium carotovorum* subsp. *carotovorum*, *Fusarium sambucinum*, *Phytophthora infestans*, *F. graminearum*, and *Sclerotinia sclerotiorum* (Somalraju et al., 2022). Additionally, Se enhances plant resistance to pathogen invasion by improving photosynthetic efficiency, maintaining cellular and organelle integrity, and reducing oxidative stress (**Figure 3**). Moreover, Se can damage the conidial plasma membrane, disrupt osmotic regulation, and impair cellular integrity, ultimately inhibiting the mycelial growth of fungal pathogens (**Figure 3**).

For instance, Se has been shown to protect the photosynthetic apparatus under pathogen stress by increasing chloroplast size and restoring chloroplast ultrastructure in rape leaves (Filek et al., 2010). Moreover, Se application significantly reduced mitochondrial membrane permeability, lesion size, and the incidence of Sclerotinia stem rot caused by *Sclerotinia sclerotiorum* in rape (Xu et al., 2020). Se suppresses *S. sclerotiorum* by inhibiting mycelial growth, disrupting sclerotial ultrastructure, reducing antioxidant enzyme activity and acid production, and increasing ROS accumulation within fungal tissues, which ultimately limits sclerotia formation and germination in oilseed rape (Cheng et al., 2019). However, Se significantly inhibited spore germination and germ tube elongation of *Botrytis cinerea* in tomato (Wu et al., 2014, Wu et al., 2015). Similarly, Se supplementation impaired spore germination, germ tube elongation, membrane integrity, and mycelial growth of *Penicillium expansum* (Wu et al., 2014). Moreover, foliar application of Se during fruit development effectively reduced gray mold incidence in tomato by enhancing antioxidant defense responses (Zhu et al., 2016). Higher Se concentrations restricted fungal growth, proliferation, and aflatoxin production in *Aspergillus flavus* (Zohri et al., 1997; Pacheco and Scussel, 2007). Se also induces hyphal abnormalities and structural distortions, delays the growth of *Fusarium graminearum*, reduces colony diameter, and significantly suppresses deoxynivalenol accumulation (Li et al., 2003; Mao et al., 2020). Furthermore, Se application inhibited the growth of *Fusarium oxysporum* and significantly decreased the incidence of wilt symptoms in tomato plants (Companioni et al., 2011).

### 6.2 Se-mediated defense against insect pests

**Figure 4:**
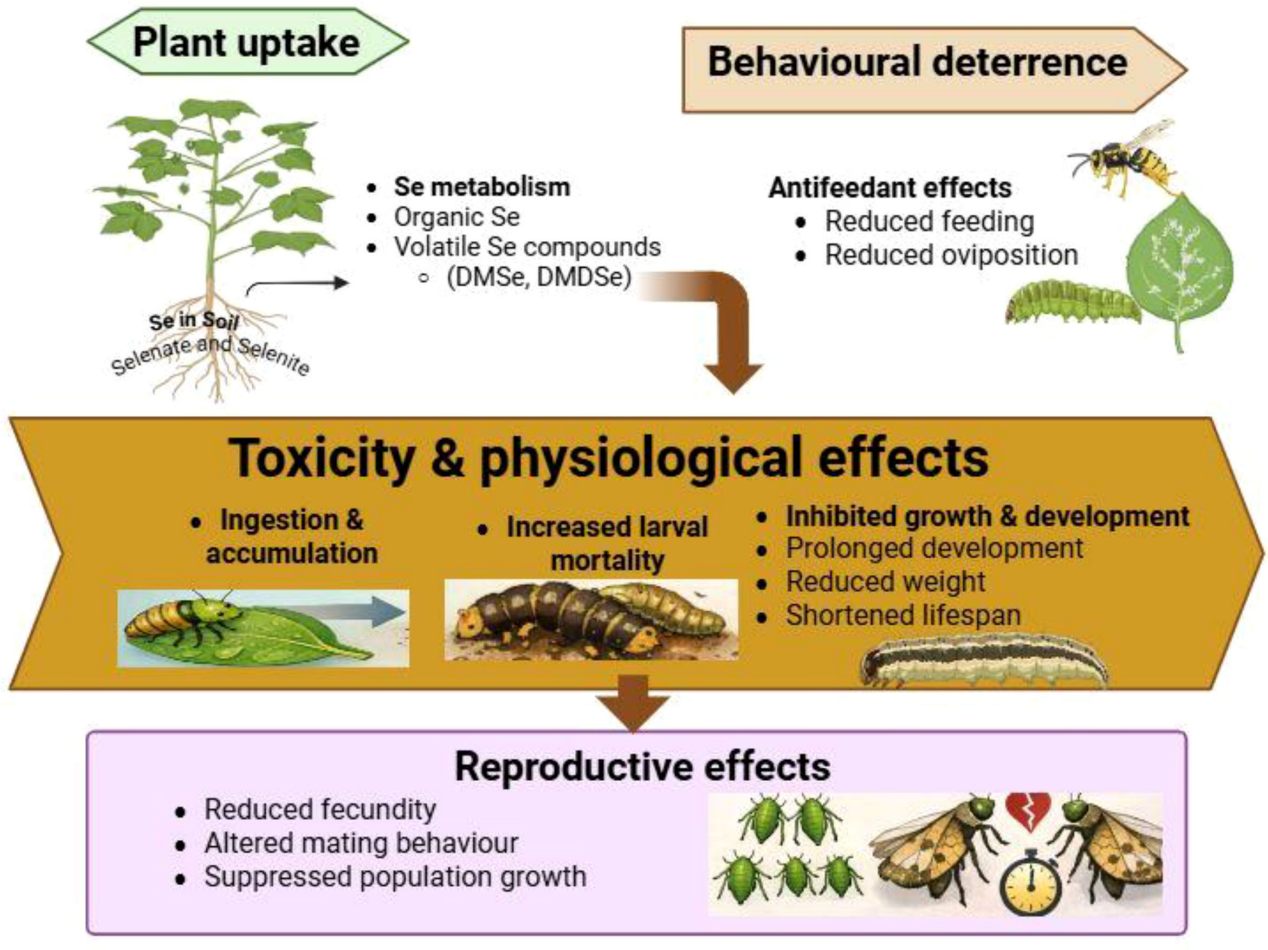
Mechanism of Se-mediated plant defense against insect pests. Plants absorb Se from soil and convert it into organic and volatile compounds, including dimethyl selenide (DMSe) and dimethyl diselenide (DMDSe). These compounds contribute to behavioral deterrence by reducing insect feeding and oviposition. Se accumulation induces toxicity and physiological effects, including increased larval mortality and inhibited growth and development. Additionally, Se negatively affects reproduction by reducing fecundity, altering mating behavior, and suppressing population growth.

Se is considered one of the earliest investigated systemic pesticides for pest control, as indicated by early reports (Gnadinger, 1933; Reed et al., 1962). Numerous studies have examined the effects of Se on insect pests feeding on Se-accumulating plants (Maier and Knight, 1993; Trumble et al., 1993; Hanson et al., 2004; Franz et al., 2011). Selenium enters the food chain when plants absorb it from the soil and can be transferred across trophic levels, including to insects that feed on these plants (Scheys et al., 2020; So et al., 2023). Plants can metabolize accumulated Se into volatile compounds such as dimethyl selenide (DMSe) and dimethyl diselenide (DMDSe) (Franz et al., 2011; Li et al., 2023), which act as natural insect repellents and disrupt feeding behavior and oviposition in insect pests (**Figure 4**). Moreover, elevated Se concentrations in plant tissues exert direct toxic effects on certain insect species (Franz et al., 2011; So et al., 2023), resulting in increased mortality, reduced reproductive rates, inhibited growth and development, and shortened adult lifespan (**Figure 4**).

Insect pests are affected by Se in a dose-dependent manner, and due to its high-water solubility, Se can be effectively applied in solution- and diet-based toxicity assays (Li et al., 2023; So et al., 2023). Both inorganic (selenate and selenite) and organic (selenomethionine and selenocysteine) forms exhibit variable toxicity across different insect taxa (Scheys et al., 2020; So et al., 2023). It has been demonstrated that a Se-enriched diet alters plant selection and feeding behavior, acting as an antifeedant for larvae of *Spodoptera exigua* (Trumble et al., 1998; Vickerman and Trumble, 1999). Similarly, inorganic Se showed stronger antifeedant effects than organic Se compounds against late-instar larvae of *S. exigua* (Vickerman et al., 2002). Notably, selenate acts as a strong feeding deterrent, whereas organic Se compounds are less commonly associated with pest deterrence; however, volatile biogenic Se compounds such as DMSe and DMDSe may contribute to pest repellence (Terry and Zayed, 1998). Choice-feeding experiments have shown that at lower Se concentrations, crickets prefer Se-containing plants, whereas higher concentrations significantly deter feeding (Freeman et al., 2007). Similarly, in *Pieris rapae*, larvae prefer Se-deficient leaves and exhibit higher feeding rates compared with Se-enriched foliage (Hanson et al., 2003). The rate of infestation by *Myzus persicae* on mustard plants was significantly higher in Se-deficient plants, approaching 100% after one week compared with Se-treated plants (Hanson et al., 2004). Furthermore, SeNPs demonstrated notable toxicity against *Spodoptera litura* larvae, with antifeedant activity (Arunthirumeni et al., 2022).

The effects of Se on insect antibiosis have been widely documented. For example, increasing concentrations of selenate or selenite prolonged the developmental duration of *S. exigua* to pupal and adult stages (Trumble et al., 1998). Elevated Se levels in leaves have been *negatively correlated with population growth of Myzus persicae* on mustard (Hanson et al., 2004). Similarly, *S. litura* larvae feeding on Se-treated plants exhibited a reduction in body weight (Popham et al., 2005). High Se concentrations also inhibited the growth and development of *Ostrinia furnacalis*, as evidenced by reduced eclosion rates, lower pupal weights, decreased longevity, and extended pupal duration (Han et al., 2017). The body size and reproductive capacity of adult moths emerging from *S. exigua* larvae fed on Se-treated plants were significantly reduced (Rothschild, 1969). Similarly, Se negatively affected the reproduction of the peach aphid (*Myzus persicae*), and at 4.2 mg kg^-^¹, reduced the fecundity of *Chironomus triangulifer* (Conley et al., 2011). In addition, *Ostrinia furnacalis* females reared on artificial diets containing 75 mg kg^-^¹ Se showed reduced courtship duration and delayed mating behavior (Han et al., 2017). The population growth of *M. persicae* declined by Se, while higher concentrations caused mortality (Hanson et al., 2004). Newly hatched *P. rapae* larvae fed on plants containing 1300 mg kg^-^¹ Se died within 9 days, whereas older larvae succumbed within 2 days when exposed to 1600 mg kg^-^¹ Se (Hanson et al., 2003). Furthermore, exposure of *Nilaparvata lugens* nymphs to 10.6 μM sodium selenite resulted in over 80% mortality within three days, highlighting the direct toxic effects of Se on insect pests (Scheys et al., 2020).

### 6.3 Mechanistic overview of SeNPs-mediated tolerance against biotic stress

SeNPs play a vital role in modulating tolerance against biotic stress via both direct antimicrobial effects and indirect activation of plant defense mechanisms (Cheng et al., 2025). By adhering to the cell walls of pathogenic microbes, SeNPs may restrict the proliferation of phytopathogens by causing membrane disruption, intracellular leakage, and ultimately cell death. Their nanoscale size enhances antimicrobial efficacy by increasing the surface area available for interaction with microorganisms (Cheng et al., 2025). Furthermore, SeNPs generate ROS that disrupt proteins, lipids, and nucleic acids, interfere with DNA and RNA synthesis, inhibit key enzymes involved in cellular respiration, and ultimately suppress pathogen growth (Burmistrov et al., 2025; Cheng et al., 2025).

In addition to direct antimicrobial toxicity, SeNPs enhance plant defense through induced systemic resistance (ISR). It has been demonstrated in several studies that SeNPs stimulate antioxidant enzymes such as glutathione peroxidase, ascorbate peroxidase, and superoxide dismutase, while increasing the accumulation of secondary metabolites, including flavonoids and phenolics involved in plant defense (Nag et al., 2024). These compounds act as antimicrobial phytoalexins, restricting pathogen invasion and strengthening plant tissues. Soil or foliar application of SeNPs has been demonstrated to reduce disease severity in crops by activating defense enzymes and key enzymes of the phenylpropanoid pathway, such as phenylalanine ammonia-lyase (PAL), which are crucial for plant responses against biotic stress (Burmistrov et al., 2025; Narang et al., 2025).

The crosstalk among phytohormone signaling pathways such as ethylene (ET), jasmonic acid (JA), and salicylic acid (SA) is closely associated with SeNP-mediated activation of plant defense responses (Shang et al., 2023). JA and ET signaling pathways are associated with defense against necrotrophic pathogens and insect pests, whereas SA signaling is primarily linked to systemic acquired resistance (SAR) and resistance against biotrophic phytopathogens (Wei et al., 2024). It has been found that SeNPs increase the endogenous levels of JA and SA, thereby inducing the expression of defense-related genes, including pathogenesis-related proteins, and enhancing systemic resistance (Chen et al., 2024). Plants are able to restrict pathogen invasion and disease progression through the coordinated interaction of these hormonal pathways, while fine-tuning immune responses (Shang et al., 2023; Nag et al., 2024; Narang et al., 2025).

## 7 Integration of multi-omics technologies in Se research

The complex molecular mechanisms underpinning Se-induced stress tolerance are being increasingly elucidated through advanced multi-omics technologies. The integration of genomics, transcriptomics, proteomics, and metabolomics enables a comprehensive systems-level understanding of how selenium and selenium nanoparticles regulate plant defense responses, moving from observational phenotyping to a detailed mechanistic framework of stress tolerance (Wang et al., 2023c; Dong et al., 2025; Zhang et al., 2025b).

### 7.1 Genomics and epigenomics

Genomics and epigenomics provide the foundational framework for understanding the heritable potential and regulatory shifts underpinning plant responsiveness to selenium. While genome-wide association studies (GWAS) have traditionally identified loci for traits like drought resistance in crops such as rice and sorghum (Guo et al., 2018; Spindel et al., 2018), future GWAS studies could be extended to identify genetic variants associated with selenium uptake, utilization efficiency, and metabolism, thereby facilitating the breeding of Se-enriched and stress-resilient cultivars. SeNPs enhance plant stress resilience by upregulating aquaporin genes and key ion transporter genes, such as *NHX1, CAX1, SOS1*, and H^+^-ATPase, which regulate cellular water balance and ion homeostasis under stress conditions. This coordinated regulation of K^+^/Na^+^ balance promotes efficient water transport and strengthens plant tolerance to salinity stress in wheat (Soliman et al., 2023). Furthermore, epigenomics explores heritable modifications beyond the DNA sequence. Techniques like whole-genome bisulfite sequencing have quantified stress-induced DNA methylation changes in crops such as maize and soybean (Liang et al., 2019; Long et al., 2021), providing a framework to investigate how selenium priming induces beneficial epigenetic modifications that enhance stress memory and adaptive responses. These insights are crucial for developing selenium-efficient cultivars and understanding the long-term protective effects of Se applications.

### 7.2 Transcriptomics

Transcriptomics directly reveals the gene expression reprogramming induced by Se and selenium nanoparticles that underlies stress tolerance. It has been demonstrated that exogenous Se application enhances drought tolerance in *Nicotiana tabacum* by modulating the expression of drought-responsive microRNAs (miRNAs), which regulate key target genes, including *reduced wall acetylation 2*, *extensin-1-like*, *cation/calcium exchanger 4-like*, *serine/threonine protein phosphatase 2A*, and *squamosa promoter-binding-like protein 4*, involved in cell wall modification and stress signaling (Dai et al., 2023). Moreover, exogenous application of Se upregulates genes associated with photosynthetic machinery, glutathione metabolism, phenylpropanoid biosynthesis, hormone signaling, and MAPK signaling pathways, thereby improving redox balance and metabolic adaptation under stress conditions in alfalfa (Wang et al., 2025). Similarly, transcriptomic analyses have consistently revealed the upregulation of genes involved in ROS scavenging and antioxidant defense mechanisms (Zeeshan et al., 2023; Liu et al., 2024; Deng et al., 2025b). For instance, in rice under salt stress, SeNPs priming has been shown to regulate the glutathione cycle, a key pathway involved in ROS detoxification and stress signaling (Xing et al., 2024). Furthermore, this approach demonstrates that selenium nanoparticles modulate the expression of transcription factors and other stress-responsive genes, thereby facilitating adaptive responses to environmental challenges (Daler et al., 2024; Xing et al., 2024; Qin et al., 2025). This provides a molecular-level framework for understanding the activation of defense pathways responsible for enhanced stress tolerance.

### 7.3 Proteomics

Proteomics extends insights from gene expression to functional protein dynamics involved in Se-mediated stress responses. Proteomic analyses have identified significant changes in protein profiles associated with stress response pathways, including signal transduction and protein folding mechanisms, which are essential for maintaining cellular function under stress conditions (Zhu et al., 2024; Wang et al., 2025). This approach has been used to show that SeNPs enhance the abundance of key antioxidant enzymes in salt-stressed wheat (Soliman et al., 2023). Deeper insights can be gained through phosphoproteomics, which reveals how selenium modulates signaling pathways by altering the phosphorylation status of regulatory proteins (Wu et al., 2023). This functional validation confirms that transcriptional changes are effectively translated into active defense responses.

### 7.4 Metabolomics

Metabolomics captures the final biochemical output, revealing how Se treatment reprograms the plant metabolic profile and defense-related metabolites (Yang et al., 2023; Deng et al., 2025a). Metabolomic studies in tobacco have identified several stress-associated metabolites, including N-acetylneuraminic acid and catechin, and highlighted the nta-miR97-5p–LLR-RLK–catechin regulatory module as a potential mechanism underlying Se-mediated drought tolerance (Dai et al., 2023). Similarly, metabolomic studies have shown that selenium nanoparticles modulate the biosynthesis of secondary metabolites and osmolytes, which play critical roles in osmotic adjustment and stress signaling (Zhou et al., 2024; Abdelsalam et al., 2025b). This has been clearly demonstrated in case studies**;** for example, in mustard plants, SeNPs enhanced stress tolerance by increasing antioxidant enzyme activities and metabolite accumulation, as evidenced by gas chromatography–mass spectrometry analyses (Sarkar and Kalita, 2022a). A key aspect of this metabolic adjustment is the transformation of SeNPs into bioactive selenium compounds, such as selenomethionine and selenocysteine, which further support metabolic reprogramming and stress adaptation (Xing et al., 2024). This final layer of evidence directly links Se treatment to the accumulation of protective metabolites responsible for enhanced stress resilience. By integrating these multi-omics datasets, scientists can construct a comprehensive framework spanning genetic potential (genomics), transcriptional regulation (transcriptomics), protein function (proteomics), and metabolic outcomes (metabolomics). This system’s biology approach is critical for developing precision strategies for the application of selenium and selenium nanoparticles in sustainable agriculture, transforming them from general stress mitigators into targeted tools for improving crop resilience.

## 8 Toxicity and the dose-response paradigm

The application of Se in agriculture represents a classic dose–response relationship, where the boundary between a beneficial micronutrient and a phytotoxic element is remarkably narrow. The effects of Se are dual in nature, and its impact, whether positive or negative, depends critically on its chemical form, concentration, and the specific plant species (Hawrylak-Nowak et al., 2015; Schiavon and Pilon-Smits, 2017; Moulick et al., 2024).

### 8.1 Phytotoxicity of bulk selenium

At elevated concentrations, Se shifts from a beneficial agent to a phytotoxic element. Two key factors contribute to Se toxicity: (i) excessive accumulation of Se within plant cells beyond threshold levels, and (ii) competition between Se and sulfur in biochemical pathways and structural incorporation due to their chemical similarity (White et al., 2004; Tian et al., 2017; Moulick et al., 2024). Plants treated with SeO_3_²^-^ exhibit chlorosis and inhibited growth (Hasanuzzaman et al., 2020b). Moreover, Se toxicity is associated with enhanced anthocyanin accumulation in the leaves of various plants, including maize, following the application of SeMet (Hawrylak-Nowak, 2008). The symptoms of toxicity are severe and may include disruption of plasma membrane integrity, premature senescence, chlorosis, and reduced yield (Hasanuzzaman et al., 2021). Compared with mature plants, seedlings are more sensitive to Se toxicity, and SeO_3_²^-^ is considerably more toxic than SeO_4_²^-^ (Hawrylak-Nowak, 2013).

The toxicity threshold of SeO_3_²^-^ varies significantly among plant species. The maximum concentrations of SeO_3_²^-^ in the growth medium that did not inhibit plant development were reported as 10.0 mg L^-^¹ for alfalfa, 1.0 mg L^-^¹ for radish, and 0.25 mg L^-^¹ for sunflower and chard (Guevara Moreno et al., 2018). In contrast, cucumber exhibited biomass reduction at minimum concentrations of 20.0 µM (SeO_3_²^-^) and 80.0 µM (SeO_4_²^-^), whereas lettuce showed toxicity at lower concentrations (15.0–20.0 µM) (Hawrylak-Nowak, 2013; Hawrylak-Nowak et al., 2015). At the cellular level, Se disrupts protein stability and induces oxidative damage, including protein oxidation and nitration, lipid peroxidation, and alterations in cellular redox balance, as well as the formation of dysfunctional selenoproteins (Kolbert et al., 2016; Liu et al., 2022). The tolerance of agricultural plants to Se varies widely among species, even though plant tissues may accumulate more than 5.0 mg kg^-^¹ Se. For example, rice exhibits toxicity at approximately 2.0 mg kg^-^¹ Se, whereas Dutch clover can tolerate up to 330.0 mg kg^-^¹, and toxic symptoms in wheat begin to appear at around 4.9 mg kg^-^¹ (Klusoňová et al., 2015; Guevara Moreno et al., 2018; Lu et al., 2024; Moulick et al., 2024).

### 8.2 Nano-specific toxicity considerations

While SeNPs are generally recognized for their reduced toxicity and increased biocompatibility compared to bulk Se, they are not without risk and exhibit dose-dependent phytotoxic effects (Samynathan et al., 2023). Recent studies indicate that nano-Se can induce toxicity depending on concentration and plant species, similar to bulk Se forms (Babajani et al., 2019; Neysanian et al., 2020; Abedi et al., 2021). Studies have shown that both Se and nano-Se can inhibit plant growth, with reduced fruit production observed in *Cichorium intybus* L. and tomato, as evidenced by decreases in leaf number and flower production (Neysanian et al., 2020; Abedi et al., 2021). Negative effects on biochemical and growth parameters have also been reported, including reductions in NPK, Zn content, and photosynthetic pigments in *Vigna unguiculata* (Silva et al., 2018).

At the biochemical level, assessing physiological status involves monitoring key stress indicators, including phytohormone profiles (e.g., IAA, ABA, jasmonic acid), osmotic regulators (e.g., free amino acids and soluble sugars), markers of cellular integrity (e.g., crude protein and fatty acid content), and oxidative stress indicators (e.g., lipid peroxidation) (Hussein et al., 2019; Wang et al., 2020). However, while beneficial at low doses, Se can become toxic at higher concentrations due to its narrow optimal range. This toxicity often arises from disruption of the antioxidant defense system, where excessive levels of biogenic SeNPs trigger overproduction of ROS, leading to increased H_2_O_2_ accumulation and lipid peroxidation, while simultaneously suppressing key antioxidant enzymes such as POD (Fallah et al., 2024). This damage is evident at the cellular level, as demonstrated by tannic acid-capped SeNPs, which reduced chlorophyll a and b contents at concentrations of 20 mg L^-^¹ and 80 mg L^-^¹, respectively (Samynathan et al., 2023). Because excessive Se leads to severe phytotoxic symptoms, including chlorosis, premature senescence, and eventual plant death (Hasanuzzaman et al., 2020b), the application of SeNPs, despite their advantages in biocompatibility, requires careful dose optimization to maximize benefits while minimizing adverse effects for sustainable agricultural use (Samynathan et al., 2023).

## 9 Conclusions and future perspectives

This comprehensive review highlights the pivotal roles of Se and SeNPs in modulating plant defense mechanisms against a wide range of biotic and abiotic stresses. As an element with a narrow margin between benefit and toxicity, the efficacy of Se is strongly influenced by its chemical form, concentration, and plant species. We have established that Se enhances the plant antioxidant defense system by activating key enzymatic and non-enzymatic components, thereby maintaining ROS homeostasis and reducing oxidative damage. Beyond antioxidant activity, Se improves photosynthetic performance, preserves chloroplast integrity, regulates osmoprotectants, and stimulates the synthesis of protective secondary metabolites. The emergence of SeNPs represents a significant advancement, offering improved biocompatibility, lower toxicity, and enhanced bioavailability compared with bulk Se forms, making them promising candidates for biofortification and stress mitigation. The integration of multi-omics technologies is now unraveling the complex molecular networks underlying Se-mediated stress tolerance, providing a systems-level understanding of its mode of action.

Despite this progress, several critical knowledge gaps remain. A primary challenge is bridging the gap between controlled laboratory findings and field-scale applications. The long-term environmental fate, persistence, and ecological risks associated with SeNPs require comprehensive evaluation to ensure their safe and sustainable use in agriculture. Furthermore, while the phenotypic effects of Se are well documented, the underlying molecular signaling pathways responsible for initiating protective responses remain incompletely understood. The role requireepigenetic regulation, transcription factor activity, and hormone-mediated signaling pathways requires further investigation. Another limitation lies in the limited understanding of SeNPs uptake, translocation, and subcellular interactions across different plant species.

Future research should focus on several key areas to fully harness the potential of Se and SeNPs in sustainable agriculture. First, the development of standardized, scalable, and cost-effective green synthesis approaches for SeNPs is essential for their practical application. Second, long-term and large-scale field trials are necessary to validate their efficacy and safety across diverse agro-climatic conditions and crop systems. Third, integrated multi-omics approaches should be employed to elucidate Se/SeNP-mediated molecular signaling networks, which will facilitate the development of Se-efficient crop varieties and precision nano-enabled agrochemicals. Finally, there is a need to establish clear regulatory frameworks and species-specific dosage guidelines to maximize benefits while minimizing potential toxicity risks. By addressing these priorities, Se nanotechnology can be strategically advanced to mitigate the impacts of climate change on agriculture, thereby supporting food security and sustainable crop productivity.
